# Synovial Fibroblast Extracellular Vesicles Induce Inflammation via Delivering miR-21-5p in Osteoarthritis

**DOI:** 10.3390/cells14070519

**Published:** 2025-03-31

**Authors:** Vasileios Konteles, Ioanna Papathanasiou, Maria Tzetis, Anastasios Kriebardis, Aspasia Tsezou

**Affiliations:** 1 Laboratory of Cytogenetics and Molecular Genetics, Faculty of Medicine, University of Thessaly, 41222 Larissa, Greece or vkonteles@med.uoa.gr (V.K.); iopapat@uth.gr (I.P.); 2 Choremion Research Laboratory, Department of Medical Genetics, National and Kapodistrian University of Athens, 11527 Athens, Greece; mtzetis@gmail.com; 3 Laboratory of Reliability and Quality Control in Laboratory Hematology (HemQcR), Department of Biomedical Sciences, School of Health & Caring Sciences, University of West Attica (UniWA), 12243 Egaleo, Greece; akrieb@uniwa.gr

**Keywords:** extracellular vesicles, osteoarthritis, miR-21-5p, inflammation

## Abstract

Small extracellular vesicles (sEVs) derived from different osteoarthritic (OA) tissues regulate OA-related biological processes through transporting their content (proteins, miRNAs, etc.) to recipient cells. This study aimed to characterize the miRNA profile of synovial fibroblasts-derived small EVs (FS_OA_sEVs) and investigate their role in inflammation in chondrocytes. Chondrocytes were isolated from macroscopically preserved and lesioned OA cartilage (C_OAmin and C_OAmax) and synovial fibroblasts from OA synovium. Synovial fibroblasts-derived small EVs (FS_OA_sEVs) were characterized according to ISEV guidelines and used for miRNA profiling and bioinformatics analysis. miR-21-5p was identified as one of the most abundant, and its target genes, such as KLF6, were enriched in OA-related processes including inflammation. Treatment of C_OAmin chondrocytes with FS_OA_sEVs resulted in decreased expression of COL2A1 and ACAN and an increase in catabolic markers MMP-3 and MMP-13. Moreover, C-OAmin receiving FS_OA_sEVs exhibited increased levels of inflammatory markers and miR-21-5p expression, resembling chondrocytes’ phenotype from lesioned OA cartilage, whereas miR-21-5p inhibition reversed their expression of inflammatory markers and miR-21-5p. Compared to C_OA min, C_OAmax chondrocytes exhibited increased miR-21-5p and inflammatory markers expression and decreased KLF6 expression. miR-21-5p inhibition in C_OAmax led to KLF6 upregulation and suppression of inflammatory mediators, whereas co-treatment with siRNA against KLF6 negated this effect, confirming a potential direct regulatory relationship between miR-21-5p and KLF6. Our results provide novel insights into the FS_OA_sEV-mediated inflammation axis, highlighting FS_OA_sEV-derived miR-21-5p as a driver of OA progression via regulating inflammation in chondrocytes.

## 1. Introduction

Osteoarthritis (OA) is a common musculoskeletal disorder affecting over 650 million individuals worldwide [1,2]. It involves structural changes of the whole joint and is characterized by chronic pain and functional disability [3]. Multiple risk factors, including ageing, obesity, and metabolic factors along with mitochondrial dysfunction play key roles in OA pathogenesis [4]. Despite OA high prevalence and global impact, currently, there are no drugs that modify or halt OA progression and development [5]. Today, OA is considered a highly heterogenous disease consisting of multiple endophenotypes, with cartilage-driven, bone-driven, and synovial inflammation-driven phenotypes being among the major ones [6]. While osteoarthritis (OA) can affect multiple joints, the knee joint is the most commonly impacted, contributing to approximately 85% of the global load of OA [7,8]. The knee joint comprises a complex microenvironment consisting of different type of cells, such as synovial fibroblasts (FS), representing about 75% of synovium cells, as well as immune cells [9], mesenchymal stem cells, adipocytes, osteoblasts, and chondrocytes, which interact via paracrine, autocrine, and endocrine pathways to achieve joint homeostasis [10]. Maintaining homeostasis is a crucial event in the joint, which is disrupted during OA process. In that regard, an interesting study by Chou et al. (2020), using single-cell RNA-sequencing (scRNA-seq), on OA knee joint tissue cells predicted potential regulators of chondrocyte gene expression during OA progression to demonstrate the molecular cross-talk between cartilage and synovium. The authors reported on the role of pro-inflammatory cytokines, exclusively expressed by synoviocytes, as important mediators of OA chondrocytes’ phenotype, pointing towards the significant contribution of synovial cell populations in synovium inflammation and cartilage destruction in OA [11].

Furthermore, it has been shown that microRNAs (miRNAs), the endogenous small non-coding RNAs with length of approximately 20–22 nucleotides, play major role in main OA processes, such as extracellular matrix (ECM) formation, degradation, apoptosis, and inflammation [12]. miRNAs function at the post-transcriptional expression level by affecting the stability and translation of mRNA targets in parental cells as well as recipient cells when delivered by extracellular vesicles (EVs) [13].

Extracellular vesicles (EVs), an heterogeneous group of cell-derived bilipid membrane-bound particles ranging in size from 50–2000 nm [14], have been shown recently to play key role in the OA joint [15,16]. Small EVs (sEVs) (30–200 nm) are secreted in the knee OA joint by synovial fibroblasts, chondrocytes, osteoblasts, tenocytes, and other joint cells and mediate cell-to-cell communication [17,18]. Specifically, it has been shown that OA synovial fibroblasts and chondrocytes-derived sEVs are drivers of inflammation, cartilage destruction, and loss of joint function [19]. sEVs contain a rich biological cargo of mRNAs, proteins, and non-coding RNAs, including miRNAs, originating from the host cells, which are delivered in recipient cells regulating their expression [20]. Examination of miRNAs signatures in sEVs derived from synovial fluid and chondrocytes exhibited distinct expression patterns among OA and non-OA individuals, suggesting altered intercell communication within the OA joint microenvironment [21]. With advances in technology and better characterization of EVs cargo, it can be speculated that EVs from different cell sources in the OA joint will be identified as new OA indicators and will be employed as a potential therapeutic strategy for OA patients in the future.

OA FS-derived EVs were shown to carry unique miRNAs signatures compared to parental cells [22] and to regulate chondrocyte inflammatory and remodeling responses. In that regard, Wang et al. demonstrated that FS-EVs contribute to joint homeostasis by modulating the innate immune response, specifically through the delivery of miR-150-3p to neighboring chondrocytes [23]. Recently, miR-21-5p has attracted significant interest due to its involvement in bone diseases by regulating osteogenic and osteoclastogenic differentiation [23]. Specifically, in an OA mouse model, miR-21-5p was found to be enriched in apoptotic bodies of M2 macrophage-derived EVs and to alleviate OA by changing the macrophage’s phenotype [24].

In the present study, we identified, at first, the miRNAs signatures in sEVs secreted from OA synovial fibroblasts (FS_OA_sEVs) and subsequently aimed to explore the functional role of FS_OA_sEVs-derived miR-21-5p in inflammation induction in osteoarthritic chondrocytes.

## 2. Materials and Methods

### 2.1. Patients and Samples

Synovial and cartilage tissues were derived from seven (7) individuals with OA (4 females; mean age 67.4 ± 3.2 years and 3 males; mean age 69 ± 1.5 years) who underwent total knee replacement surgery at the Department of Orthopedic Surgery of University Hospital of Larissa, Greece (UHL). After surgery, the whole knee joint was sent to the Laboratory of Cytogenetics and Molecular Genetics at the same hospital, where different OA joint tissues were isolated for further experiments. Cartilage samples were harvested from the tibial plateaus and/or femoral condyles and were classified based on gross morphology as either severely damaged (OAmax) and harvested from the main lesion site subjected to the highest load or mildly damaged (OAmin) and collected from regions without visible surface damage [25]. Synovial tissues with reddish appearance were collected from the lateral or medial sides of the knee joint, transferred to sterile culture medium, sliced, and placed in culture (for approx. 5 days) to receive synovial fibroblasts (SF). Radiographs were obtained for all patients before surgical procedure and were evaluated for OA severity using the Kellgren–Lawrence (K/L) classification system. All individuals had OA with assigned K/L grade greater than 2. A blinded clinical assessment of the radiographs was performed independently by two expert clinicians. Individuals diagnosed with immune-related diseases such as rheumatoid arthritis, chondrodysplasias, and post-traumatic OA were excluded from research.

The research protocol complied with the ethical standards outlined in the 1975 Declaration of Helsinki and approved from University Hospital of Larissa Ethics Committee.

### 2.2. Chondrocytes and Synovial Fibroblasts Cultures

Chondrocytes were derived from cartilage with severe OA damage (C_OAmax) and from areas with no obvious surface defects (C_OAmin), while synovial fibroblasts were derived from OA synovium (FS_OA) [26]. Cartilage specimens and OA synovium were excised and subjected to enzymatic digestion with 1 mg/mL pronase and 1 mg/mL collagenase P (Roche Applied Science, Mannheim, Germany). Next, C_OAmax, C_OAmin, and FS _OA cells from individual specimens were separately cultured in flasks using Dulbecco’s Modified Eagle’s Medium/Ham’s F-12 (Thermo Fisher Scientific, Waltham, MA, USA), supplemented with 10% fetal bovine serum (FBS) and 1% penicillin-streptomycin (GIBCO, Thermo Fisher Scientific, Waltham, MA, USA). C_OAmax, C_OAmin, and FS_OA cell cultures were placed in a humidified atmosphere (5% CO_2_) incubator at 37 °C until they reached the desired confluence.

### 2.3. Small Extracellular Vesicles (sEVs) Isolation

Upon reaching 80% confluency, passage 1 FS_OA cultures were rinsed with phosphate-buffered saline (PBS) and culture medium containing DMEM/F12 (GIBCO, Thermo Fisher Scientific, Waltham, MA, USA) enriched with 10% exosome-depleted fetal bovine serum (FBS) and 1% penicillin-streptomycin (P/S) (for both, GIBCO, Thermo Fisher Scientific, Waltham, MA, USA) was added. After 48 h, the conditioned medium from and FS_OA cultures was collected for the isolation of small extracellular vesicles (sEVs) (50 nm–200 nm) according to recent ISEV guidelines [27], using the polyethylene glycol-precipitation method (using Total Exosome isolation kit (for cell culture media), Invitrogen, Waltham, MA, USA). Briefly, the conditioned medium was centrifuged at 300× *g* for 10 min, followed by 2000× *g* for 30 min at room temperature (RT). Using a 0.22 μm filter (Steritop, Millipore, Merck KGaA, Darmstadt, Germany), residual cells and cell debris were removed from the supernatant. Total exosome isolation reagent was then added to the filtered medium, which was incubated overnight at 4 °C. The mixture was centrifuged at 10,000× *g* for 60 min to retrieve the sEV pellet. Finally, pellet resuspension was performed in a volume of 1X PBS or the elution buffer provided with the exosome isolation kit. sEVs were stored at −80 °C for further experiments.

### 2.4. sEVs Protein Quantification and Western Blot

The protein concentration of sEVs was measured on a fluorometer (Qubit 4.0) using the Qubit Protein Assay Kit (Thermo Fisher Scientific, Waltham, MA, USA). Cell lysis was performed using a radioimmunoprecipitation assay buffer (RIPA) supplemented with 1 mM phenylmethanesulfonyl fluoride (PMSF; Beyotime Biotechnology Co., Ltd., Shanghai, China). Protein samples separation, membranes non-specific binding blocking, and primary antibody co-incubation were performed as previously described [28]. The primary antibodies used included anti-CD63 (1:1000, D4I1X, Rabbit mAb #55051S; Cell Signaling Technology, Danvers, MA, USA), anti-CD81 (1:1000; E2K9V, Rabbit mAb, #52892S; Cell Signaling Technology, Danvers, MA, USA), and anti-Calnexin (1:1000; C5C9, Rabbit mAb, #2679S; Cell Signaling Technology, Danvers, MA, USA). Following overnight co-incubation, HRP conjugated secondary antibodies (anti-Rabbit; 1:10.000, BA1054-1; Boster Biological Technology, Pleasanton, CA, USA) were incubated with membranes at room temperature (RT) for 1 h. Visualization of protein bands was performed as previously described [28,29]. All Western blots were conducted in triplicate.

### 2.5. Transmission Electron Microscopy

The morphology of FS_OA_sEVs was assessed by transmission electron microscopy (TEM). Isolated exosomes were fixed in 2% paraformaldehyde in phosphate buffer and then adsorbed on formvar-carbon coated copper grids. Afterwards, sEVs were contrasted with uranyl oxalate and embedded in a mixture of 4% uranyl acetate and 2% methylcellulose. Finally, the samples were examined by TEM.

### 2.6. Nanoparticle Tracking Analysis (NTA)

The concentration and size of FS_OA-derived sEVs were assessed by NTA using the NS300 NanoSight instrument (ATA Scientific, Caringbah, Australia), with automatic settings applied for maximum jump distance and blur to ensure optimal tracking accuracy. For this, 10 μL aliquots of each sEVs sample were diluted in 1 mL filtered PBS before analysis. Particle size and concentration were determined based on Brownian motion and diffusion coefficient, as measured by NTA. All analyses were performed in NTA 3.0 software. Measurements were performed five (5) times for each sample, and size distribution was averaged.

### 2.7. Microarrays for FS_OA_sEVs miRNA Profiling and Expression Analysis

miRNA profiling in FS_OA_sEVs was performed using Agilent miRNA microarrays (SurePrint G3 Human miRNA, Gene Expression 8 × 60 K, miRBase release 21.0, Agilent Technologies, Santa Clara, CA, USA) according to manufacturer’s protocol and as previously described [30]. Following hybridization and washing, the G2505C Agilent scanner (Agilent Technologies, Santa Clara, CA, USA) was used for arrays’ scanning. Data were normalized by quantile normalization using the “limma” package [31]. Values detected include *p*-values that assess whether each probe signal is significantly higher than *p*-values of the negative controls. Lower *p*-values indicate that the probe reflects the presence of truly expressed miRNA. The *p*-values were computed using linear models. The *p*-values adjustments were performed using the false-discovery rate (FDR) algorithm in R environment. FDR ≤ 0.05 indicates significantly expressed miRNAs.

### 2.8. Enrichment Analysis and miRNAs’ Target Genes Prediction

Enrichment analyses, focused on Gene Ontology (GO) and Kyoto Encyclopedia of Genes and Genomes (KEGG) pathways, were performed using the “enrichR” tool in R environment. *p*-value < 0.05 used as a threshold for functions and pathways that were considered as significantly enriched. The top GO terms were visualized using dot plot analysis. Target prediction for all expressed miRNAs was conducted using the TargetScan (https://www.targetscan.org/vert_80 version 8.0), miRDB (https://mirdb.org, version 6.0), and miRTarBase (https://mirtarbase.cuhk.edu.cn, version 9.0) databases. Regulatory interactions between expressed miRNAs and their predicted targets were constructed using the “network” tool in R environment and were visualized on the Cytoscape platform (version 3.9.1).

### 2.9. sEVs Labeling and Uptake Assay

FS_OA_sEVs were labeled with the PKH67 green-fluorescent kit, according to manufacturer’s instructions (Sigma-Aldrich, St. Louis, MO, USA). Upon reaching 80% confluency, C_OAmin cultures were washed twice with phosphate-buffered saline (PBS), and the medium was substituted with DMEM/F12 (GIBCO, Thermo Fisher Scientific, Waltham, MA, USA) enriched with 10% exosome-depleted fetal bovine serum (FBS) exosome-depleted fetal bovine serum (FBS) and 50 μg/mL PKH67-labeled FS_OA_sEVs. Next, cells were harvested following 24 and 48 h incubation time, and cell pellets were fixed in 2% formaldehyde solution for 20 min, followed by PBS washes to remove excess stain. For cellular localization, 4′,6-diamidino-2-phenylindole (DAPI; Sigma-Aldrich, St. Louis, MO, USA) was used for nuclei stain.

### 2.10. Scratch Assay

C_OAmin chondrocytes were seeded into 6-well plates, and gap generations were performed by making a scratch in the cell layer using a 1000 μL pipette tip. Culture medium (without serum) containing 50 μg/mL FS_OA-derived sEVs (FS_OA_sEVs) was added to the wells, while serum-free culture medium without sEVs was used as negative control. The migration of C_OAmin into the gap (visualized as black doted lines) was imaged at 0, 24 h, and 48 h, and the widths of the scratched region were measured using the ImageJ software (Wayne Rasband, National Institutes of Health, Bethesda, MD, USA).

### 2.11. Transfection Experiments of C_OAmax with miR-21-5p Inhibitor and KLF6 siRNA

C_OAmax chondrocytes were seeded onto 6-well plates containing approximately 3 × 10^5^ cells per well. Culture medium was removed after 24 h of incubation, and cells were treated for 48 h under the following conditions: (a) 25 pmol mirVana^®^ miRNA inhibitor miR-21-5p (#4464084, Thermo Fisher Scientific), (b) 25pmol siKLF6 (Hs_KLF6_1 FlexiTube siRNA, ID: SI00025074, QIAGEN) with Negative control #1 (#4464076, Thermo Fisher Scientific), and (c) 25 pmol mirVana^®^ miRNA inhibitor miR-21-5p (#4464084, Thermo Fisher Scientific) with 25pmol siKLF6 (Hs_KLF6_1 FlexiTube siRNA, ID: SI00025074, QIAGEN). Lipofectamine™ RNAiMAX reagent (Thermo Fisher Scientific, Waltham, MA, USA) was utilized for cell transfections according to the manufacturer’s instructions. Following transfection, C_OAmax chondrocytes and culture medium were collected, and RNA extraction was performed for subsequent experimental procedures.

### 2.12. Transfection of C_OAmin Chondrocytes with FS_OA_sEVs and miR-21-5p Inhibitors

C_OAmin chondrocytes were seeded into 6-well plates containing approximately 3 × 10^5^ cells per well. Culture medium was removed after 24 h of incubation, and cells were treated for 48 h under the following conditions: (a) 50 μg/mL FS_OA-derived sEVs (FS_OA_sEVs), (b) 50 μg/mL FS_OA_sEVs with mirVana^®^ miRNA inhibitor miR-21-5p Negative control #1 (#4464076, Thermo Fisher Scientific) as negative control, and (c) 50 μg/mL FS_OA_sEVs with mirVana^®^ miRNA inhibitor miR-21-5p (#4464084, Thermo Fisher Scientific). Lipofectamine™ RNAiMAX reagent (Thermo Fisher Scientific, Waltham, MA, USA) was utilized for cell transfections according to the manufacturer’s instructions.

### 2.13. RNA Extraction, cDNA Synthesis, and Quantitative Real-Time PCR (qRT-PCR)

Initially, total RNA was extracted from C_OAmax and C_OAmin chondrocytes using TRIzol reagent (Thermo Fisher Scientific, Waltham, MA, USA). Complementary DNA (cDNA) synthesis was performed using SuperScript III reverse transcriptase (Thermo Fisher Scientific, Waltham, MA, USA). miRNAs quantitative amplification was performed by target cDNA extension with a uniquely designed stem-loop primer during first-strand cDNA synthesis [31]. The expression levels of type II collagen (COL2A1), aggrecan (ACAN), matrix metalloproteinase (MMP)-3, matrix metalloproteinase (MMP)-13, miR-21-5p, transcription factor KLF6, interleukin-1β (IL-1β), interleukin-6 (IL-6), and tumor necrosis factor α (TNFα) were quantified using the ABI 7300 system (Applied Biosystems, Bedford, MA, USA). Experimental procedures and expression data analysis were performed as previously described [28]. The RT-qPCR protocol consisted of 40 cycles with a final step of 60 °C for 1 min. The oligonucleotide primers for RT-qPCR were designed using Primer3 software v.2.2.3 (IDT PrimerQuest™ Tool). Table 1 contains all primer sequences used. Glyceraldehyde 3-phosphate dehydrogenase (GAPDH) was used as internal control for mRNA expression and U6 small nuclear RNA (snRNA) for miRNA expression normalization. All reactions were performed in duplicate. Instructions of Livak et al. 2001 were used for relative gene expression analysis using 2^−∆∆Ct^ method [32].

### 2.14. ELISA Assay

Following transfections with FS_OA_sEVs and/or miR-21 inhibitor, the cell culture medium was collected followed by gentle centrifugation. The secreted cytokine concentrations of inflammation markers IL-1β, IL-6, and TNFα, were measured using three different ELISA kits (Boster Biological Technology, Pleasanton, CA, USA) for each marker (Human IL-1β (EK0392) Human TNFα (EK0525), and Human IL-6 (EK0410)). All experiments were applied following the manufacturer’s instructions. Cell supernatants’ inflammatory cytokine concentrations were calculated using the four-parameter logistic (4-PL) curve-fit model.

### 2.15. Statistical Analysis

SPSS 25.0 software used for all data analysis. The Shapiro–Wilk normality test was assessed for data distribution. Unpaired/paired *t*-test, Mann–Whitney U-test or Wilcoxon matched-pairs test were used, where appropriate, for statistical significance determination. Results are presented as mean ± standard error, and *p*-value < 0.05 indicates their statistical significance. All reactions for qRT-PCR experiments were performed in duplicate, using a minimum of three (3) OA samples unless otherwise specified.

## 3. Results

### 3.1. Isolation and Characterization of Small EVs Derived from OA Synovial Fibroblasts

**Figure 1:**
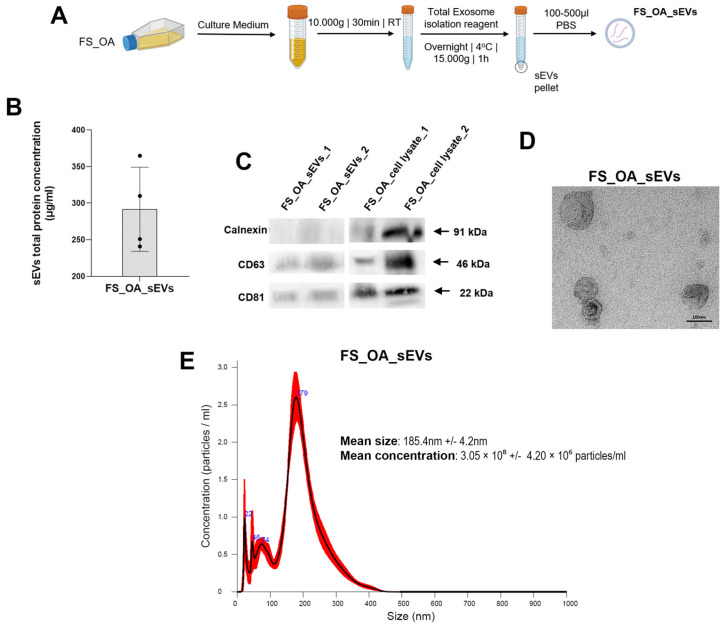
Characteristics of sEVs derived from OA synovial fibroblasts (FS_OA_sEVs). (**A**) Protocol of FS_OA_sEVs isolation using the polyethylene glycol-precipitation method. (**B**) Quantification of FS_OA_sEVs. (**C**) Positive sEVs markers CD81 and CD63 detected in both FS_OA_sEVs samples; calnexin marker exclusively present in cell lysates. (**D**) TEM images of sEVS (scale bars: 100 nm). (**E**) NTA, mean particle size of sEVs (measured five times): mean particle size for FS_OA was 185.4 ± 4.2 nm and mean particle concentration/mL 3.05 × 10^8^ ± 4.20 × 10^6^.

Chondrocytes derived from cartilage with advanced OA lesions (C_OAmax) and from regions with macroscopically not visible OA lesions (C_OAmin) as well as synovial fibroblasts (FS_OA) obtained from the synovium were cultured. Then, FS_OA culture medium was collected for isolation of small extracellular vesicles (sEVs) using the polyethylene glycol-precipitation method (Figure 1A). sEVs characterization encompassed surface markers quantification, morphological assessment, and size distribution analysis. To confirm the identity of the isolated sEVs, Western blot analysis of three protein markers (Calnexin, CD63, and CD81), followed by transmission electron microscopy for shape identification and nanoparticle tracking analysis for size distribution were performed. In addition, the total protein concentration of FS_OA_sEVs was quantified, yielding a value of 289 ± 0.19 μg/mL. (Figure 1B). Moreover, the positive sEVs markers CD81 and CD63 were detected in FS_OA_sEVs, while the negative marker Calnexin was exclusively present in the cell lysate of FS_OA and absent in the sEV samples (Figure 1C). TEM analysis revealed that the majority of sEVs from FS_OA exhibited a characteristic spherical bilayer membrane structure of approximately 100 nm diameter (Figure 1D). NTA further demonstrated that 80–90% of the particles were within the expected size range of sEVs (50–200 nm), while the mean particle size of FS_OA_sEVs was 185.4 ± 4.2 nm and mean particle concentration 3.05 × 10^8^ ± 4.20 × 10^6^ particles/mL (Figure 1E).

### 3.2. FS_OA_sEVs Affect Chondrocytes’ Anabolic and Catabolic OA Gene Markers and Reduce Cell Motility

**Figure 2:**
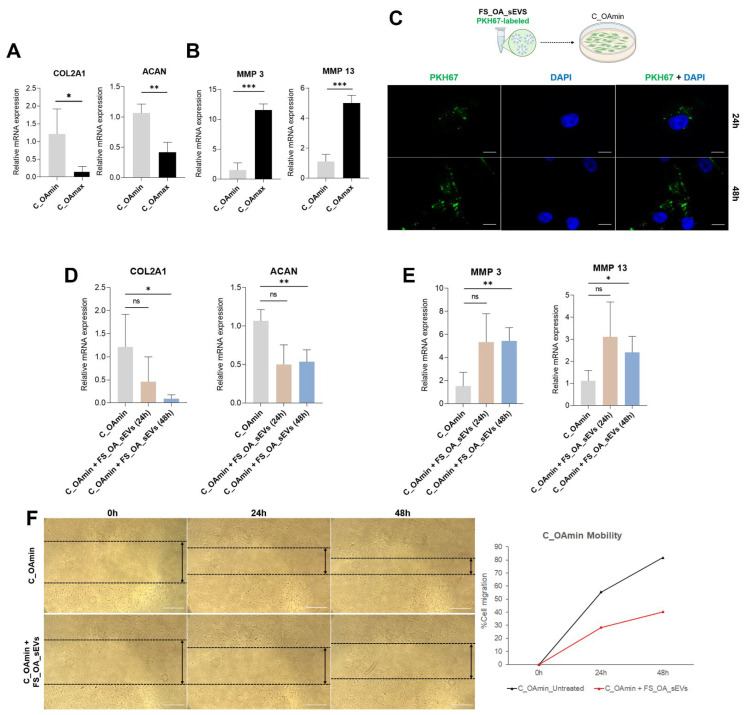
FS_OA_sEVs increase the expression of OA anabolic markers and reduce cell mobility in C_OAmin chondrocytes. (**A**,**B**) C_OAmin chondrocytes exhibit significant increase in COL2A1 and ACAN and decrease in MMP-3, and MMP-13 mRNA expressions compared to C_OAmax (*n* = 7). (**C**) PKH67-labeled FS_OA_sEVs after 24 and 48 h co-culture with C_OAmin reveal green fluorescence at chondrocytes’ perinuclear region (scale bar = 50 μm). (**D**,**E**) Treatment of C_OAmin chondrocytes with FS_OA_sEVs results in increased expression of anabolic markers and significantly reduced expression of catabolic markers (*n* = 5). (**F**) Images from scratch assay experiment at three different time points. C_OAmin and C_OAmin + FS_OA_sEVs were plated into 6-well plates and wounded with a pipette tip, and migration into the gap (visualized as two parallel dotted black lines) was measured with ImageJ software. * *p* < 0.05; ** *p* < 0.01; *** *p* < 0.001; ns = no significance.

To investigate the possible uptake of FS_OA-secreted sEVs by chondrocytes and the impact on the osteoarthritic phenotype of chondrocytes, FS_OA_sEVs were stained with PKH67 fluorescent dye and were subsequently co-cultured for 24 and 48 h with C_OAmin, which were characterized by elevated expression of anabolic markers (COL2A1 and ACAN) and reduced expression of catabolic markers (MMP-3 and MMP-13) compared to C_OAmax chondrocytes (Figure 2A,Β), resembling the phenotype of normal chondrocytes. Co-culturing of C_OAmin with PKH67-labeled FS_OA_sEVs for 24 h revealed green fluorescence at the region surrounding the chondrocyte nucleus, confirming endocytosis of PKH67-labeled sEVs by C_OAmin cells. Fluorescence intensity within the chondrocytes progressively increased over time and reached saturation at 48 h, highlighting the efficient uptake and high absorption of FS_OA_sEVs by chondrocytes (Figure 2C). Furthermore, treatment of C_OAmin chondrocytes with FS_OA_sEVs resulted in decreased expression of anabolic markers COL2A1 and ACAN (Figure 2D) and significantly increased expression of the catabolic markers MMP-3 and MMP-13 (Figure 2E), enhancing their osteoarthritic phenotype. Additionally, using scratch assay procedure, it was observed that the width of the gap in the 6-well plate containing C_OA min + FS_OA_sEVs was bigger than the gap containing C_OAmin, indicating FS_OA_sEVs reduced the mobility of C_OAmin chondrocytes (Figure 2F). The above results indicated that FS_OA_sEVs may play a significant role in regulating OA progression.

### 3.3. miRNA Pattern of FS_OA_sEVs

**Figure 3:**
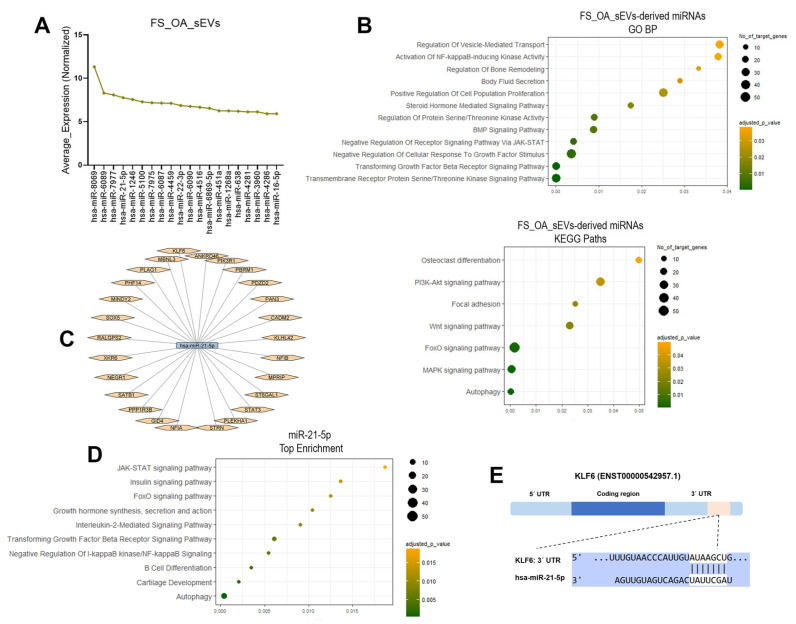
FS_OA_sEVs miRNAs landscape. (**A**) Top 20 expressed miRNAs in FS_OA_sEVs. (**B**) Dot plot with −log10 (p-adjusted) indicating GO BP terms and KEGG paths of the top 50 expressed FS_OA_sEVs-derived miRNAs. (**C**) miR-21-5p predicted mRNA targets. (**D**) GO terms and KEGG paths of miR-21-5p mRNA targets visualized by dot plot with −log10. (**E**) Schematic representation of a predicted miR-21-5p binding site in KLF6 mRNA 3′UTR.

To explore the role of sEVs-derived miRNAs in OA onset and progression, we next evaluated the expression profile of miRNAs in sEVs isolated from FS_OA using miRNA microarrays and found that 291 miRNAs were expressed in FS_OA_sEVs (Supplementary Table S1). miR-8069, miR-6089, miR-7977, miR-21-5p, and miR-1246 were demonstrated to be highly expressed in FS_OA_sEVs (Figure 3A). To investigate the potential roles of the top 50 miRNAs expressed FS_OA_sEVs, their mRNA targets were identified using the TargetScan, miRTarBase, and miRDB databases (Supplementary Table S2). Gene Ontology (GO) enrichment and KEGG pathway analyses of the top 50 expressed miRNAs in FS_OA_sEVs revealed their involvement in key processes related to inflammation and autophagy, including tight junction formation, control of cell migration and proliferation, regulation of bone remodeling, and promotion of cell-to-matrix attachment (Figure 3B).

Following sEVs-derived miRNA analysis, miR-21-5p was chosen for further evaluation of its role in OA progression, as miR-21-5p has been previously found to be associated with osteoarthritis progression [33,34]. Enrichment analysis of the predicted miR-21-5p mRNA targets (Figure 3C) revealed their involvement in critical processes associated with OA progression, such as cartilage development, autophagy, inflammation, and modulation of key OA signaling pathways, including JAK-STAT, FoxO, and NF-κB (Figure 3D). Among the predicted target genes, the transcription factor KLF6, with a pivotal role in the regulation of inflammation, was selected for further experimental procedures (Figure 3E).

### 3.4. FS_OA_ sEVs-Derived-miR-21-5p Induces Inflammation-Related Genes in C_OAmin Chondrocytes

**Figure 4:**
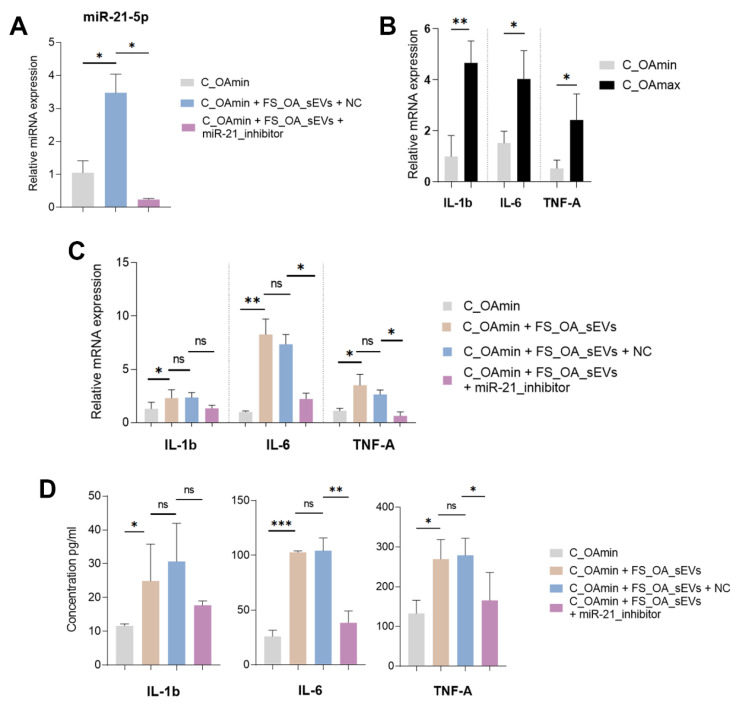
FS_OA_sEVs-derived miR-21-5p delivery into C_OAmin induces inflammation. (**A**) miR-21-5p exhibits significantly increased expression in FS_OA_sEV-treated chondrocytes compared to untreated cells; co-treatment with miR-21-5p inhibitor results in marked reduction in its expression (*n* = 5). (**B**) C_OAmin chondrocytes exhibit low expression of inflammatory markers (IL-1β, IL-6, and TNF-α) compared to C_OAmax (*n* = 7). (**C**,**D**) IL-1β, IL-6, and TNF-α gene expression and cytokine secretion levels are reduced in FS_OA_sEVs/miR-21-5p inhibitor-treated group compared to FS_OA_sEVs-only group (*n* = 5). * *p* < 0.05; ** *p* < 0.01; *** *p* < 0.001; ns = no significance.

To investigate the role of FS_OA_sEV-derived miR-21-5p in the inflammatory state of chondrocytes, C_OAmin chondrocytes were treated with FS_OA_sEVs with or without miR-21-5p inhibitor, and the expression levels of key inflammatory genes (IL-1β, IL-6, and TNF-α) were assessed. C_OAmin chondrocytes treatment with FS_OA_sEVs plus miR-21-5p inhibitor led to an expected reduction in miR-21-5p expression compared to FS_OA_sEVs/NC (Figure 4A). Before treatment, C_OAmin chondrocytes exhibited low mRNA expression levels of inflammatory markers (IL-1β, IL-6, and TNF-α), whereas C_OAmax chondrocytes displayed an inversely proportional inflammatory expression profile (Figure 4B). In addition, on FS_OA_sEV-treated cells, IL-1β, IL-6, and TNF-α mRNA expression levels (*p* = 0.0488, *p* = 0.097, and *p* = 0.0472, respectively) and cytokine secretion levels (*p* = 0.025, *p* = 0.0002, and *p* = 0.043, respectively) were significantly upregulated compared to untreated C_OAmin chondrocytes. However, co-treatment with miR-21-5p inhibitor resulted in a reduction in both mRNA expression levels (*p* = 0.0601, *p* = 0.0137, and *p* = 0.0370) and cytokine secretion levels (*p* = 0.0508, *p* = 0.0053, and *p* = 0.0461) compared to the FS_OA_sEVs-only group (Figure 4C,D).

### 3.5. FS_OA_ sEVs-Derived miR-21 Inhibits KLF6 Expression and Promotes IL-1β, IL-6, and TNF-α Expressions in C_OAmax Chondrocytes

**Figure 5:**
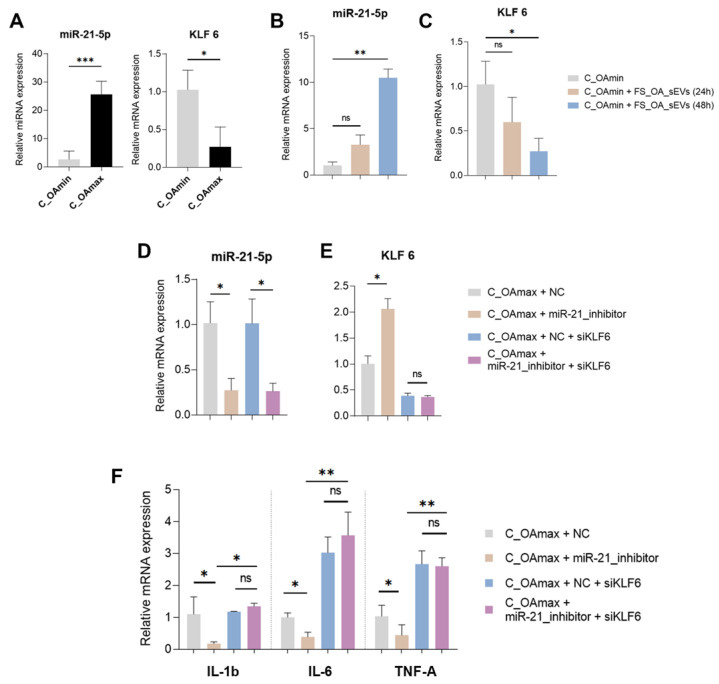
miR-21 promotes inflammation in C_OAmax chondrocytes via KLF6 inhibition. (**A**) C_OAmin chondrocytes exhibit decreased miR-21-5p and increased KLF6 mRNA expression compared to C_OAmax (*n* = 7) (**B**,**C**) Treatment of C_OAmin chondrocytes with FS_OA_sEVs leads to increase in miR-21-5p expression and decrease in KLF6 mRNA expression. (**D**,**E**) Successful miR-21-5p suppression after miR-21-5p inhibitor (*n* = 4); C_OAmax chondrocytes treated with miR-21-5p inhibitor show significant increase in KLF6 expression compared to negative inhibitor-treated cells (*n* = 4). (**F**) IL-1β, IL-6, and TNF-α mRNA expression levels are significantly reduced compared in C_OAmax chondrocytes to NC-treated cells; miR-21-5p inhibitor/siKLF6-treated C_OAmax show significantly increased IL-1β, IL-6, and TNF-α expression levels compared to miR-21-5p inhibitor-only cells (*n* = 4). * *p* < 0.05; ** *p* < 0.01, *** *p* < 0.001; ns = no significance.

Firstly, we evaluated miR-21-5p and KLF6 expression in C_OAmin and C_OAmax chondrocytes and found that C_OAmin chondrocytes exhibited decreased miR-21-5p and increased KLF6 mRNA expression compared to C_OAmax (Figure 5A). Interestingly, treatment of C_OAmin chondrocytes with FS_OA_sEVs for 48 h led to increase in miR-21-5p expression and significant decrease in KLF6 mRNA expression, suggesting that FS_OA_ sEVs effectively deliver miR-21-5p into C_OAmin chondrocytes, modulating KLF6 expression (Figure 5B,C).

To further validate whether miR-21-5p-mediated inhibition of KLF6 directly influences OA-associated inflammatory markers, C_OAmax chondrocytes were transfected with a miR-21-5p inhibitor either alone or in combination with siRNA targeting KLF6. Following miR-21-5p inhibitor treatment and the subsequent successful suppression of miR-21-5p expression (Figure 5D), C_OAmax chondrocytes exhibited an increased KLF6 expression compared to miRNA-inhibitor negative control (NC)-treated cells (Figure 5E). Interestingly, in miR-21-5p inhibitor-treated C_OAmax chondrocytes, where KLF6 expression levels were restored, IL-1β, IL-6, and TNF-α mRNA expression levels were markedly decreased compared to NC-treated cells. However, in the miR-21-5p inhibitor/siKLF6-treated group, significantly increased expression in IL-1β and IL-6 was observed compared to the miR-21-5p inhibitor-only group (Figure 5F), suggesting that miR-21-5p directly regulates KLF6 expression, thereby influencing the expression of inflammatory mediators.

## 4. Discussion

Recent evidence has pointed out that EVs released by different type of cells of the OA joint mediate intercellular communication via delivering their biological cargo, especially miRNAs, to target cells [16]. sEVs have been demonstrated to play an important role in the inflammation process, enhancing local or systemic inflammation due to stimulation of inflammatory cytokine production, such as IL-1β, IL-6, and IL-8 [35], in recipient cells. Furthermore, miRNAs associated with sEVs have been shown to regulate inflammatory processes [36] by either enhancing or suppressing inflammatory reactions [37].

In the present study, at first, we fully characterized, in line with current ISEV guidelines (2023) [38], the small EVs secreted from OA synovial fibroblasts (FS_OA_sEVs) and investigated their impact on the osteoarthritic phenotype of chondrocytes. Co-culture of chondrocytes obtained from regions with no signs of OA damage (C_OAmin) with PKH67-labelled FS_OA_sEVs revealed increased fluorescence intensity at the chondrocytes’ perinuclear region, confirming FS_OA_sEVs uptake by chondrocytes. Moreover, treatment of C_OAmin chondrocytes with FS_OA_sEVs led to decreased expression of anabolic markers COL2A1 and ACAN and increased catabolic markers MMP-3 and MMP-markers compared to no treatment, enhancing the OA phenotype of chondrocytes obtained from regions with low signs of osteoarthritis. These findings are in agreement with a previous in vivo study reporting that synovial tissue-derived EVs induce the expression of cartilage degradation-related enzymes in normal chondrocytes in an OA rabbit model [39]. Similarly, Ashgar S. et al. recently reported that in vitro sEVs derived from synovial fibroblasts contribute to chondrocyte damage in OA [22]. Besides the impact of FS_OA_sEVs on the catabolic function of chondrocytes, FS_OA_sEVs were also shown to negatively affect the migration ability of C_OAmin chondrocytes, as evidenced by the scratch assay. It is known that chondrocytes’ migration from healthy to injured tissues is one of the most important challenges during cartilage repair [40]. C_OAmin chondrocytes after the uptake of FS_OA_sEVs were converted to a pathological state resembling chondrocytes from advanced OA lesions, resulting in reduced migration to cartilage defects and subsequently reduced ability for cartilage repair.

Strong evidence supports that sEVs secreted from OA synovial fibroblasts carry miRNAs that can regulate cartilage catabolic responses by targeting genes related to OA onset and progression [22].

To explore the role of sEVs-derived miRNAs in OA processes, we proceeded by evaluating the miRNAs profile of sEVs from FS_OA and found 291 miRNAs to be highly expressed in FS_OA_sEVs; among the top highly expressed ones were miR-21-5p, miR-22-3p, and miR-16-5p. A relative study reported that miRNAs signatures of sEVs derived from OA synovial fibroblasts differed from parental OA synovial fibroblasts and found that miR-4472-2, miR-1302-3, miR-6720, miR-182, miR-6087, and miR-4532 were the most significantly enriched in comparison to parental cells [22].

We previously demonstrated that miR-22 expression in chondrocytes is highly correlated with body mass index (BMI), and inhibition of its expression ameliorated the inflammatory and catabolic state of osteoarthritic chondrocytes, demonstrating miR-22 as an OA-related miRNA [41]. Regarding miR-16-5p, Li et al. demonstrated that upregulated miR-16-5p promoted the catabolic function of chondrocytes and suppressed the expression of ECM genes by targeting SMAD-3 [42]. In contrast, in an in vitro OA model, increased expression of miR-16-5p attenuated chondrocyte dysfunction by inactivating MAPK signaling, indicating the dual role of miR-16-5p in OA progression [42].

Enrichment analysis of genes targeted by FS_OA_sEVs-derived miRNAs revealed their involvement in key OA processes related to inflammation and autophagy, including modulation of cell migration and movement and promotion of cell-matrix attachment. Although osteoarthritis inflammation is a complex process, it is considered a central driver of OA progression, affecting multiple cells and tissues in an OA joint [43,44]. Moreover, dysfunction of autophagy has been correlated with disrupted function of miRNAs in OA, contributing to cartilage damage [45]. In our study, we highlight the involvement of miRNAs derived from OA synovial fibroblasts in main OA processes leading to cartilage degradation, highlighting the synovium–cartilage cross-talk in OA development.

MiRNA profile analysis revealed miR-21-5p as the fourth most abundant miRNA in FS_OA_sEVs. MiR-21-5p has recently gained attention, as it has been shown to have a key role in inflammation in OA animal models. Specifically, miR-21-5p was found to be enriched in apoptotic bodies of M2 macrophage-derived EVs and to alleviate OA in an OA mouse model by reversing the inflammatory response caused by M1 macrophages [24]. Another in vivo study using a surgical rat OA model reported that extracellular miR-21 was the most abundant among increased miRNAs in the synovial tissue and mediated knee OA pain [34] through TLR7 activation. To investigate the effect of FS_OA_sEV-derived miR-21-5p in the inflammatory state of chondrocytes, C_OAmin chondrocytes were treated with FS_OA_sEVs with or without miR-21-5p inhibitor. We found that FS_OA_sEV-derived miR-21-5p regulated key inflammatory markers IL-1β, IL-6, and TNF-α expression, driving C_OAmin chondrocytes towards an inflammatory pathological state and enhancing OA progression.

Enrichment analysis of miR-21-5p-predicted target genes revealed their involvement in critical processes associated with OA progression, such as cartilage development, autophagy, inflammation, and modulation of key signaling pathways, including JAK-STAT, FoxO, and NF-κB. Interestingly, among miR-21-5p targets was the transcription factor Krüppel-like factor 6 (KLF6), a widely expressed nuclear transcriptional regulator [46] that was previously identified by our group, using integrative miRNA and proteomic approaches, as a novel OA protein downregulated in OA chondrocytes [41]. Recently, Liu C. [47], using weighted gene co-expression network analysis (WGCNA), found significant gene modules associated with OA and among them identified KLF6 as an autophagy-related gene. KLF6 expression has been shown to be cell-type-dependent and associated with the pathogenesis of multiple diseases. In vivo and in vitro studies have demonstrated KLF6 implication in different pathologies, such as cardiac fibrosis [48] as well as hepatic and renal fibrosis [49]. Increased KLF6 expression has been also associated with inflammatory diseases such as IBD [50] and psoriasis [51] and with enhancement of inflammation in macrophages. Furthermore, KLF6 has been shown to activate collagen a1(I) promoter and TGF- promoters, establishing a link between KLF6 activity and cytokine responsiveness to injury [52].

To investigate a potential regulatory interaction between FS_OA_sEV-derived miR-21-5p and KLF6 in OA chondrocytes, C_OAmax chondrocytes were treated with miR-21-5p inhibitor, and KLF6 expression levels were found to be restored, while inflammatory cytokines’ IL-1β, IL-6, and TNF-α mRNA expression levels were significantly reduced compared to negative control-treated cells. However, miR-21-5p inhibitor/siKLF6-treatement resulted in increased IL-1β, IL-6, and TNF-α mRNA expression levels in comparison to the miR-21-5p inhibitor-only group, suggesting that miR-21-5p directly regulates KLF6 expression, thereby promoting the expression of inflammatory mediators.

A regulatory relationship between MSC-EVs-derived miR-21-5p and KLF6 has been recently shown in atherosclerosis and diabetes, diseases that are both characterized by metabolic deregulation and inflammation, as is OA [53,54,55]. Specifically, MSCs-derived exosomes containing miR-21-5p were shown to reduce macrophage infiltration by targeting KLF6 and ERK1/2 signaling pathways, attenuating the development of atherosclerosis [56]. In addition, MSCs-EVs delivery of miR-21-5p was shown to promote skin fibroblast proliferation and migration by downregulating KLF6, pointing to a possible therapeutic strategy for diabetic foot ulcers [57].

Our study has the advantage of using chondrocytes obtained from two different cartilage regions: one exhibiting mild signs of OA damage (OAmin) and another from regions with severe OA damage (OAmax). This allowed the demonstration of the impact of FS_OA_sEV-derived-miR-21-5p in chondrocytes with minimal OA signs, providing new insights into the sEVs–inflammation axis in OA. In conclusion, our novel findings highlight the functional implication of miR-21-5p derived from synovial fibroblasts small extracellular vesicles in inflammatory processes as major contributor to cartilage destruction and OA progression.

## Figures and Tables

**Table 1:** Primer sequences and stem-loop sequences for qRT-PCR.

Gene Name	Forward Primer (5′-3′)	Reverse Primes (3′-5′)
COLII A1	ATGACAATCTGGCTCCCAACACTGC	GACCGGCCCTATGTCCACACCGAAT
ACAN	TGAGGAGGGCTGGAACAAGTACC	GGAGGTGGTAATTGCAGGGAACA
MMP 3	CCTCAGGAAGCTTGAACCTG	GGAAACCTAGGGTGTGGAT
MMP 13	TGGCATTGCTGACATCATGA	GCCAGAGGGCCCATCAA
IL-1β	ATGGACAAGCTGAGGAAGATG	CCTCGTTATCCCATGTGTCG
IL-6	CAACCTGAACCTTCCAAAGATG	ACCTCAAACTCCAAAAGACCAG
TNF-A	CCTGAAAACAACCCTCAGA	AAGAGGCTGAGGAACAAGCA
GAPDH	GAGTCAACGGATTTGGTCGT	GACAAGCTTCCCGTTCTCAG
KLF6	CAAGGGAAATGGCGATGCCT	CTTTTCTCCTGTGTGCGTCC
hsa-miR-21-5p	GCGGCAACACCAGTCGATG	TGCGTGTCGTGGAGTC
U6	GCTTCGGCAGCACATATACTAAAAT	CTCACACCGTGTCGTTCCA
	**Stem-loop primer sequences**	
has-miR-21-5p	5′-GTCGTATCCAGTGCGTGTCGTGGAGTCGGCAATTGCACT-3′	
U6	5′-CACGGAAGCCCTCACACCGTGTCGTTC-3′	

## Data Availability

Data are contained within the article and Supplementary Materials. Data are available upon request to the corresponding author.

## Supplementary Materials

The following supporting information can be downloaded at https://www.mdpi.com/article/10.3390/cells14070519/s1.
